# The Relation of Intemperance to Insanity

**Published:** 1888-01

**Authors:** Clark Bell

**Affiliations:** New York


### THE RELATION OF INTEMPERANCE TO INSANITY.*

BY CLARK BELL, ESQ.

MOREL says:

"The principal causes of insanity are: Intemperance and neglect of the laws of health." He believed that there are few of the unfavorable conditions of human life that by themselves cause more human degeneration than the excessive use of alcohol.

Dr. C. H. Hughes, the able editor of the *Alienist and Neurologist*, in speaking of the causes of insanity, says:

"The families of intemperate parents furnish the recruiting ground for insane asylums."

These unfortunate children, if not idiots or epileptics, are liable to grow up with querulous, explosive tempers, with feeble powers of self-guidance, weak in temptation, unstable, self indulgent, vicious and hysterical.

* Read July 6, 1887, before the International and Colonial Congress of Inebriety, at London, England, and September 14, 1887, before the Medico-Legal Society of New York.—Medico-Legal Journal.

Th. H. Kellogg, M. D., one of our most intelligent students of insanity, with a valuable experience, says:

"It is estimated that twenty-five per cent. of modern cases of insanity are due to the direct or indirect effects of the abuse of alcoholic stimulants."

This assertion is made with reference to the immediate effect of alcohol on individuals that consume it; and if its remote, baneful influences through generations are to be taken into account, it would be necessary to add considerably to the numerical strength of the statement.

The same authority, speaking of the Etiology of Insanity of this class or type, says:

"It is active first in exciting attacks to which there is already an hereditary or an acquired tendency, secondly: in the development *de novo* of characteristic types of mental derangement, and directly in the transmission of weak and irritable nervous systems that predispose to mental disorder."

"The types of mental aberration caused directly are:

1. Gradual physical degeneration, that attains finally to the complete extinction of the moral powers.
2. Ordinary attacks of mania and melancholia.
3. Delirium tremens.
4. Alcoholic Dementia and
5. Dipsomania.

This latter form has a double origin and includes two very different classes of cases. In the one class it results from a susceptibility to alcohol that is most intense, and inherited from intemperate parents, so that on slight indulgence the patient every now and then loses all self-control, and goes through a reckless alcoholic debauch.

In the other class the dipsomaniac tendency is simply one of the morbid impulses that occur in periodical insanity, and it will be found on close observation that these cases present insane peculiarities during the interim between the drinking bouts."

Lunier has probably furnished the most complete and reliable statistics, upon this subject, of the modern authors.

He claims that while the consumption of alcohol nearly doubled in France in the twenty years from 1849 to 1869, the cases of insanity from intemperance had risen from fifty-nine per cent. with men, and fifty-two per cent. with women. These statistics are remarkable as showing the relative increase in those districts of France where alcoholic or distilled spirits took the place of wine.

M. Lunier claims distinctly that the number of suicides everywhere in France followed the increased consumption of alcohol. He also shows that in the north of France, where the consumption of alcohol had nearly doubled in twenty years (1849 to 1869) the cases of insanity from alcohol had quadrupled among men, who consume it, without any corresponding increase among the women who did not.

He also estimates that at least fifty per cent. of the idiots and imbeciles of the great cities were the children of notorious drunkards, and that the majority of children born of parents who were constitutional drunkards are, as he considers, "weak in some way or other."

The Earl of Shaftesbury, in his evidence before the select committee of the English Parliament, in 1859, expressed his opinion that fifty per cent. of the cases admitted to English asylums were due to drink. (Evidence before Parliamentary Commission, 1859.)

While many have doubted the accuracy of this statement, it must not be lost sight of:

1. That no man in England had better or half as good facilities of observation, or would be less likely to exaggerate this per centum, than the Chairman of the Board of Lunacy Commissioners: and

2. That he doubtless took into account the cases due to intemperance as an indirect cause, and the intemperance of parents as causing insanity and idiocy in their offspring.

Bucknill & Tuke place in their list of causes of insanity, arranged in the order of their frequency and importance, intemperance as the larger and greater cause.

They verify their views by statistical evidence from the French, English and Scotch reports of the Commissioners of Lunacy, as well as the carefully prepared statements and tables of Skaie, at Morningside; Dr. Needham, at York Lunatic Asylum; Dr. Clouston, at Cumberland and Westmorland, and Drs. Kirkbride, Lee and Earle, in American Institutions.

Sir W. C. Ellis, M. D., Superintendent of Wakefield Asylum, England, in his treatise on Cause and Cure of Insanity (1838), recognizes intemperance as a prolific cause of insanity.

Samuel Wilkes, M. D., in his lectures on diseases of the Nervous System, says:

"Certainly one of the causes most frequently instrumental in the production of an atrophy of the brain is drunkenness, or the excessive use of alcoholic drinks."

Alcohol causes a degeneration of all the tissues of the body, its direct effect on the liver is cirrhosis, on the kidneys, Bright's disease, and fatty degeneration of the tissues thus caused explains the frequent death of the drunkard by heart disease.

Dr. Wilkes compares the degeneration of the tissues from the excessive use of alcohol to those processes which take place naturally in excessive old age. He says, "a drunken man is literally living too fast."

The post mortem, says the same high authority, of a confirmed drunkard, shows that the brain is shrunken and weighs so many ounces less than it did when healthy.

The legitimate effect upon the brain of the excessive use of alcohol is to produce atrophy of that organ.

Whatever arrests normal action, interferes with function and leads or tends to decay or degeneration.

Dr. Henry Maudsley gives a chapter "concerning degeneration" in his admirable treatise, "Body and Will," in which he defines degeneration, as he calls it, as an "unkinding, or the undoing of a kind."

The abuse of the use of alcohol produces a degradation of the organ by shrinking it from its normal conditions, in a lower, lesser, or baser shape, and while alcoholic atrophy of the brain is not the degradation of which Maudsley so splendidly treats, it resembles it, in physical results, in the life and health of the sufferer.

Prof. John J. Reese regards intemperance as one of the chief causes of idiocy,

Dr. William W. Ireland, a high authority upon idiocy, places heredity foremost among causes of idiocy, and does not agree with the authorities that place intemperance so high as a cause of idiocy—while he concedes that it is an important cause.

He says of drunkenness in the parents: "The children of drunken parents in many cases have an unhealthy nervous system; they are weak, unhealthy and excitable, and often have a diseased craving for spirituous liquors, but, in my opinion, idiocy is not the ordinary legacy which drunkards leave to their children."

Dr. Langdon Doun lays great stress upon intoxication of the parent at conception as a fruitful cause of idiocy.

Toussenel advances similar views.

Ludwig Dahe, the Norwegian writer on Insanity, is of the opinion that to the abuse of brandy, especially in the fathers, but also in the mothers, during pregnancy, may be assigned an important influence in the production of the large number of idiots in Norway.

The Connecticut Commission of Idiocy, in a report made to the Legislature of Connecticut in 1856, reported that the parents of idiots were given to intemperance in seventy-six cases out of two hundred and thirty-five. In thirty of these cases both parents were intemperate; in forty-six cases one parent so.

T. S. Clouston, M. D., in his able lectures delivered before the students at the University at Edinburgh, speaking of causes of insanity, says: "It is unfortunately the most common of all causes of the disease; in some cases producing it *de novo*, in others bringing into activity hereditary and acquired brain weakness."

He assigns alcohol as a cause, in whole or in part, in from fifteen to twenty per cent. of the cases of mental diseases, taking all of Scotland as a basis.

Moreau De Tours, whose studies of human degeneracy, like those of his countryman Morel, have thrown more light upon these topics than any of our English-speaking writers, agrees that the use of alcohol is one of the greatest factors working in the degeneration and deteriroation of the human species, when measured by its past results upon the race.

These degenerations are transmitted, by laws of heredity, from father to son, and seem unable to be corrected or eradicated.

Their manifestations are insanity, idiocy, ugliness, deformity, incapacity for higher education, lack of self-control, and what we might denominate the excrescences upon the normal human stock.

Its manifestations are degeneration of human tissue, reproduced and intensified, which are rarely ever cured, modified or overcome.

Dr. Edward C. Mann says: "Alcohol eventually will have to be dealt with as a source of terrible mora and physical deterioration. The whole human race is deteriorated by the poison, morally, mentally and socially. The disease of inebriety is transmitted hereditarily, causing insanity, epilepsy and idiocy, or a proclivity to crime."

Prof. Stanford E. Chaillé, Tulane University, of Louisiana, says: "The most frequent and important lesions found in the bodies of dead drunkards are of the same nature as that produced by old age. Fibroid, fatty and chalky degenerations, gradually invade the tissues and unfit them to maintain health and life. By these degenerations vital force is prematurely lowered and exhausted, and the constitution is so impaired that there is far less power to resist disease, so that it is notorious that the abusers of alcoholics greatly increase liability to death, by accidental injuries, and by epidemic and other diseases, and that, if these fail to shorten their lives, decrepitude, old age and death come on before their time.

Dr. T. D. Crothers, a very high authority, says: "It is a fact beyond question that alcohol in excess produces changes of brain circulation and nutrition, also vaso-motor paralysis, with congestion and derangement of the heart's action and diminution of the quality of the blood. The result is that both brain and nerve functions are impaired, and the capacity to realize the nature and consequence of conduct and thoughts is lessened—the victim is actually and literally on the road to insanity. The inebriate cannot be sane, for his brain is physiologically and pathologically changed."

Dr. William G. Stevenson, a high authority and a most careful and analytical observer, in speaking of the neurotic origin of criminality, says:

"That inebriety, insanity and crime bear close relationship to, and as results of pathological conditions of nerve tissue, are corelative of each other.

"It is a fact that inebriety, insanity and criminality are hereditary and undergo mutual metamorphoses during stages of transmission."

"It is a fact that drunkenness or dipsomania is a physical disease, depending on some molecular change of nerve tissue, as the direct effect of alcoholic poisoning, and the "gemmules" of this tissue, when transmitted, become active factors in the formation of character."

The same high authority says: "It is an admitted fact that inebriety in the parent predetermines insanity in the offspring."

Dr. Dodge, the late superintendent of the Binghampton Asylum for Inebriates, says:

"It is important to keep in view that drunkenness is as often involuntary as voluntary; that the person afflicted with the tendency to it obeys a law of his members more potent than his will."

Dr. Rush reports the case of an habitual drunkard who, when urged to leave off drink replied:

"Were a keg of rum in one corner of a room, and were a cannon constantly discharging balls between me and it, I could not refrain from passing before that cannon in order to get rum."

Macnish, in his *Anatomy of Drunkenness*, cites the case of a man to whom a friend painted "the distress of his family, the loss of his business and character, and the ruin of his health," who replied: "Alas, yes, this is too true! but I can no longer resist temptation; if a bottle of brandy stood at one hand and the pit of hell yawned at the other, and I were convinced that I would be pushed in as sure as I took one glass, I could not refrain."

Dr Howe, of Massachusetts, claimed that out of 300 idiots investigated by himself, one hundred and forty-five were descended from intemperate parents.

The mission of the English Society for the Study and Cure of Inebriety is of the highest importance. If, by your labors, you can benefit the future of humanity by a better understanding of the causes that have led, and are tending, to the degeneracy of mankind; if, with such knowledge, you can, in any sensible and practical way, arrest or lessen the ills of Inebriety, you will have accomplished a great good.

The study of Inebriety, and the cure of it, are now attracting world-wide attention. It is recognized by science as a disease; but has the popular mind and heart so recognized and conceded it?

Before legislation can lend its strong hand to defend the body politic, by preventive measures, based on the safety and preservation of the people from avertable ills, we must see to it that the public intellect is abreast with scientific research on the subject. This, if I understand it correctly, is the mission of the British Society and its co-laborers in America and upon the continent, and to aid your work and to hold up your hands in Great Britain, has inspired this contribution, which I hope may be for good.

